# Personalized exercise therapy during targeted drug therapy tapering in patients with rheumatoid arthritis: a pilot randomized controlled trial

**DOI:** 10.1186/s41927-026-00646-8

**Published:** 2026-05-13

**Authors:** Shinsuke Yamada, Akira Onishi, Takumi Imai, Ryuji Uozumi, Hirotaka Yamada, Kenichiro Hata, Yonsu Son, Kosuke Ebina, Yasutaka Okita, Ryota Hara, Ryu Watanabe, Tadashi Okano, Masaki Katayama, Wataru Yamamoto, Yohei Oshima, Hiroki Tanaka, Hidenori Arai, Motomu Hashimoto

**Affiliations:** 1 Department of Clinical Immunology, Graduate School of Medicine, Osaka Metropolitan University, 1-4-3-13F, Asahi-machi, Abeno-ku, Osaka City, Osaka 545-8585 Japan; 2 Department of Advanced Medicine for Rheumatic Diseases, Graduate School of Medicine, Kyoto University, Kyoto, Japan; 3 Department of Clinical and Translational Research Center, Kobe University Hospital, Hyogo, Japan; 4 Department of Industrial Engineering and Economics, Tokyo Institute of Technology, Tokyo, Japan; 5 Department of Rheumatology and Clinical Immunology, Kobe University Graduate School of Medicine, Hyogo, Japan; 6 Division of Clinical Immunology, Osaka Medical and Pharmaceutical University, Osaka, Japan; 7 First Department of Internal Medicine, Kansai Medical University, Osaka, Japan; 8 Department of Orthopaedic Surgery, The Osaka University Graduate School of Medicine, Osaka, Japan; 9 Department of Sports Medical Biomechanics, The Osaka University Graduate School of Medicine, Osaka, Japan; 10 Department of Respiratory Medicine and Clinical Immunology, The Osaka University Graduate School of Medicine, Osaka, Japan; 11 Department of Orthopaedic Surgery, Nara Medical University, Nara, Japan; 12 Center for Senile Degenerative Disorders (CSDD), Osaka Metropolitan University Graduate School of Medicine, Osaka, Japan; 13 Department of Rheumatology, Osaka Red Cross Hospital, Osaka, Japan; 14 Department of Health Information Management, Kurashiki Sweet Hospital, Okayama, Japan; 15 Rehabilitation Unit, Kyoto University Hospital, Kyoto, Japan; 16 National Center for Geriatrics and Gerontology, Obu, Aichi Japan

**Keywords:** Exercise therapy, Rheumatoid arthritis, Health-related quality of life, Physical activity

## Abstract

**Background:**

Exercise therapy (ET) is a valuable adjunctive treatment for rheumatoid arthritis (RA). We hypothesized that ET supports maintenance of remission or low disease activity (LDA) during targeted drug therapy (TT) tapering. This pilot randomized controlled trial evaluated the feasibility, safety, and preliminary effects of individualized ET in RA patients undergoing TT tapering.

**Methods:**

Thirty-two RA patients initiating TT tapering were randomized to receive personalized ET plus usual care (intervention group, *n* = 16) or usual care alone (control group, *n* = 16) for 16 weeks. The primary exploratory outcome was the proportion of patients maintaining remission or LDA (DAS28-ESR < 3.2) by week 16 without TT re-escalation. Secondary outcomes included DAS28-ESR and its component measurements at weeks 8 and 16, health-related quality of life at week 16 (SF-12), physical activity (IPAQ), and daily step count. Safety and adherence were also assessed.

**Results:**

All participants in the intervention group completed the 16-week ET program with high adherence. Remission or LDA was maintained in 75.0% (12/16) of patients in the intervention group and 81.3% (13/16) in the control group, with no clinically meaningful between-group difference. Although DAS28-ESR scores were comparable, a transient increase in physician visual analog scale was identified in the intervention group at week 8, which returned to the baseline by week 16. Over the 16-week period, IPAQ scores and step count tended to increase after adjustment for baseline values, whereas SF-12 mental health scores declined in the intervention group. No serious adverse events were observed.

**Conclusions:**

In this pilot randomized controlled trial, individualized ET during TT tapering was safe and feasible. Nevertheless, transient increases in disease activity and declines in mental health indicate the need for careful clinical monitoring and psychological support. The study was not designed to evaluate efficacy outcomes and no clinically meaningful differences between the groups in remission maintenance were identified. Larger trials will be necessary to evaluate the efficacy and long-term safety of ET during TT tapering.

**Supplementary Information:**

The online version contains supplementary material available at 10.1186/s41927-026-00646-8.

## Introduction

Exercise therapy (ET) is an important adjunctive treatment for patients with rheumatoid arthritis (RA). It has been shown to complement pharmacological as well as surgical intervention by improving physical function, reducing comorbidity risk, and enhancing quality of life (QOL) [1–3]. Exercise induces the release of myokines from skeletal muscle, which exert anti-inflammatory and metabolic regulatory effects [4–6]. Several studies have reported improvements in RA disease activity following ET [7, 8], while regular physical activity is known to be associated with a reduced risk of RA onset [9]. These findings indicate that ET may help sustain RA remission. On the other hand, inappropriate exercise can exacerbate disease activity and cause joint damage [10], highlighting the need for individualized programs tailored to disease status. In addition, initiation of ET may be associated with psychological or subjective burdens, which can influence both adherence and perceived benefits, particularly during treatment modification.

Recent advances in targeted drug therapy (TT), including biologics and Janus kinase inhibitors, have dramatically improved RA outcomes. However, long-term TT use is associated with an increased risk of infection and substantial medical costs, underscoring the recommendation for tapering once remission or low disease activity (LDA) is achieved [11]. Nonetheless, symptom flares often occur during tapering [12, 13], which highlights the need for strategies to maintain disease control.

A pilot randomized controlled trial was conducted to evaluate whether the anti-inflammatory and regulatory effects of ET could facilitate successful TT tapering. The study assessed the feasibility, safety, and preliminary effects of individualized ET on disease activity in RA patients who had achieved remission or LDA during tapering.

The primary aim of this pilot study was to assess feasibility rather than efficacy. Specifically, recruitment, retention, adherence to the prescribed exercise program, and safety (including adverse events and disease flares), along with preliminary estimates of variability in disease activity outcomes were evaluated over a 16-week period. These findings provide useful information for the design and sample size calculation of a future adequately powered definitive trial.

## Methods

### Study design and setting

This study was designed as a multicenter, parallel-group, two-arm, assessor-blinded, randomized controlled pilot trial with a 1:1 allocation ratio. It was performed in cooperation with eight universities and public hospitals in Japan that are members of the ANSWER cohort [14], including Osaka Metropolitan University, Kyoto University, The Osaka University, Osaka Medical and Pharmaceutical University, Kansai Medical University, Kobe University, Nara Medical University, and Osaka Red Cross Hospital. Patient recruitment occurred from October 19, 2022 to March 31, 2023.

The protocol complied with the Declaration of Helsinki and written informed consent was obtained from all participants by their attending physicians. Study data were collected and managed using the Research Electronic Data Capture (REDCap) system [15] hosted at Osaka Metropolitan University. An overview of participant enrollment, allocation, interventions, assessments, and visit schedules is presented in Supplementary Table 1.

### Participants

Eligible participants were adults aged 18 to 85 years diagnosed with RA classified according to the 2010 ACR/EULAR criteria [16]. Each had initiated tapering of TT after achieving remission or LDA within three months prior to baseline. To ensure appropriate understanding of the study procedures and questionnaires, all were able to communicate in Japanese.

Exclusion criteria included inability to stand or walk without support, conditions deemed inappropriate for exercise by the attending rheumatologist (e.g., pacemaker use), cognitive dysfunction at baseline, or any other condition judged as making participation inappropriate by the principal investigator or co-investigators.

### Definition of TT tapering

TT tapering was defined as a reduction in dose to less than two-thirds of the approved regimen or extension of the dosing interval to more than 1.5 times the standard interval. Specific details regarding tapering procedures for each therapy are presented in Supplementary Table 2. All modifications during follow-up, including dose re-escalation, discontinuation of tapering, and addition of concomitant therapies, were prospectively recorded.

### Intervention

Individualized ET programs were designed by professional physical therapists, based on modifications of previously established exercise protocols [6, 17]. Each program consisted of four core exercises: knee raising, standing hip abduction, heel raising, and squatting, which were performed at home for 30 to 60 minutes per session, three non-consecutive days per week [18].

Exercise intensity was individually adjusted using the Borg Rating of Perceived Exertion scale [19], with a target perceived exertion level of 5–6 (“hard”). A physical therapist tailored exercise selection, range of motion, and repetition counts based on baseline joint condition, pain level, and functional capacity.

Guidance for the ET program was provided remotely via telephone by trained research assistants under the supervision of physical therapists, with follow-up calls every four weeks to monitor adherence and, if needed, adjust intensity. Adherence was assessed using participant exercise logs and structured interviews, including number of completed exercise sessions and attainment of the target Borg RPE level. Both the intervention and control groups continued to receive regular care for RA throughout the study.

Daily physical activity was evaluated using a pedometer to record weekly step count and the International Physical Activity Questionnaire (IPAQ). Participants in the intervention group were encouraged to increase their daily step count by approximately 20% at week 8 and again at week 16 as compared with the baseline.

### Outcomes

The primary outcome was proportion of patients who maintained remission or LDA, defined as DAS28-ESR < 3.2, at week 16 without treatment re-escalation. Secondary outcomes included changes in RA disease activity assessed by DAS28-ESR at weeks 8 and 16. Additional secondary outcomes assessed at week 16 included physical disability based on the Health Assessment Questionnaire-Disability Index (HAQ-DI) [20], health-related quality of life determined with the Short Form-12 survey (SF-12) [21, 22] and EuroQol-5D (EQ-5D) [23], fatigue evaluated using the Functional Assessment of Chronic Illness Therapy-Fatigue (FACIT-F) scale [24], and physical activity shown by weekly step counts using a pedometer [25] and IPAQ scores [26].

Adverse events potentially related to study participation, such as pain, falls, fractures, orthostatic hypotension, headache, or dizziness, were recorded at each outpatient visit and through participant self-reporting. The attending physicians were also asked to monitor and document any adverse events during each follow-up appointment.

### Randomization

Following enrollment, the participants were centrally randomized to the intervention or control group using the REDCap system with a 1:1 allocation ratio. Permuted block randomization was stratified by age (≥60 vs. <60 years), sex, and baseline disease activity (DAS28-ESR ≥ 2.6 vs. <2.6). The outcome assessors were blinded to group allocation throughout the study period, whereas participants and treating physicians were not.

### RA treatments

Decisions regarding RA treatment were made solely by each attending rheumatologist according to their clinical judgment. Participants were encouraged to maintain their current tapered TT regimen during the study period, though were allowed to discontinue tapering if disease activity became difficult to control. Any other changes in RA treatment were recorded in the REDCap system and incorporated into outcome analyses conducted at each assessment point.

### Statistical analysis

In this exploratory pilot study, designed primarily to assess feasibility and safety, and also generate preliminary estimates for future trials, no formal sample size determination or power calculation was performed. Although no formal progression criteria were prespecified at trial initiation, feasibility of progression to a future definitive trial was deemed feasible if adherence exceeded 80%, retention was ≥ 85%, and no serious exercise-related adverse events occurred. These thresholds were applied descriptively to support planning of a subsequent larger trial.

Categorical variables are presented as numbers and percentages, and continuous variables as mean values with standard deviations. All statistical analyses were performed on an intention-to-treat basis. For both treatment groups, descriptive analyses were conducted at each visit for all outcomes and changes from baseline. Continuous outcomes were analyzed using mixed-effects models for repeated measurements incorporating treatment group, time, and treatment-by-time interaction, with adjustment for baseline values of the corresponding outcomes. The between-group risk difference in the proportion of patients maintaining remission or LDA was estimated with 95% confidence intervals.

Given the exploratory nature of this pilot study, no adjustments were made for multiple comparisons and results should be regarded as hypothesis-generating. Analyses were performed using the SAS software package, version 9.4 or later (SAS Institute, Cary, NC), and the R software package, version 4.2.0 or later (R Foundation for Statistical Computing, Vienna, Austria).

## Results

### Patient enrollment

**Fig. 1:**
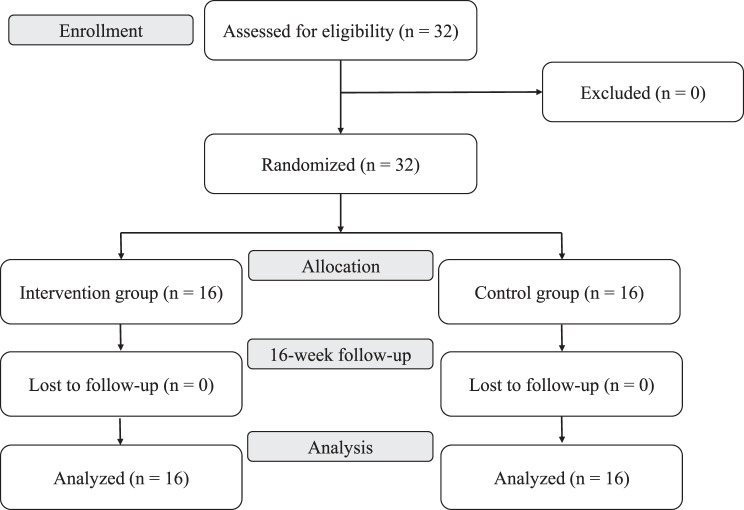
CONSORT flow diagram of participant progress through the trial. Participant flow from screening and randomization to follow-up and analysis is shown

A total of 32 patients were enrolled and randomly assigned in a 1:1 ratio to the intervention (exercise) or control group. Baseline characteristics, including baseline disease activity and comorbidities, were similar between the groups (Table 1). The flow of participants throughout the trial is shown in Fig. 1.

**Table 1:** Baseline characteristics of enrolled RA patients

	All patients	Intervention group	Control group
No.	32	16	16
Age, years	65 ± 10	65 ± 11	69 ± 8
Female gender, no. (%)	26 (81)	13 (81)	13 (81)
BMI, kg/m^2^	22.8 ± 2.8	22.8 ± 3.3	22.8 ± 2.4
RA duration, months	149 ± 121	124 ± 105	175 ± 134
ACPA positive, no. (%)	30 (94)	14 (88)	16 (100)
RF positive, no. (%)	29 (91)	14 (88)	15 (94)
TNFi, no. (%)	13 (41)	7 (44)	6 (38)
IL-6i, no. (%)	10 (31)	4 (25)	6 (38)
CTLA4-IgG, no. (%)	6 (19)	3 (19)	3 (19)
JAKi, no. (%)	3 (9)	2 (12)	1 (6)
MTX, no. (%)	15 (47)	10 (62)	5 (31)
GC, no. (%)	3 (9)	0 (0)	3 (19)
Never smoked, no. (%)	21 (66)	11 (69)	10 (62)
Past smoker, no. (%)	10 (31)	5 (31)	5 (31)
Current smoker, no. (%)	1 (3)	0	1 (6)
Hypertension, no. (%)	11 (34)	5 (31)	6 (38)
Dyslipidemia, no. (%)	11 (34)	6 (38)	5 (31)
Diabetes, no. (%)	4 (12)	3 (19)	1 (6)
Arrhythmia, no. (%)	3 (9)	2 (12)	1 (6)
Chronic heart failure, no. (%)	0	0	0
Chronic lung disease, no. (%)	1 (3)	0	1 (6)
Chronic kidney disease, no. (%)	0	0	0
Liver disease, no. (%)	0	0	0

Data are expressed as mean ± SD or number (%). BMI: body mass index, RA: rheumatoid arthritis, ACPA: anti-citrullinated protein/peptide antibody, RF: rheumatoid factor, TNFi: tumor necrosis factor inhibitor; IL-6i: interleukin-6 inhibitor; CTLA: cytotoxic T-lymphocyte antigen; JAKi: Janus kinase inhibitor; MTX: methotrexate, GC: glucocorticoid

### Effects of personalized exercise therapy on disease activity

At week 16, 75% (12/16) of the participants in the intervention group maintained remission or LDA, compared with 81.3% (13/16) in the control group. The risk difference was −6.3% (95% CI, −34.8 to 22.3) and the risk ratio was 0.92 (95% CI, 0.64 to 1.33). In both groups, mean DAS28-ESR scores remained stable at weeks 8 and 16, with minimal and clinically negligible differences between them (Table 2).

**Table 2:** Between-group comparisons of changes in disease activity, physical activity, and health-related quality of life

		Intervention group	Control group	Group difference (95% CI)	P value	Standardized effect size
DAS28-ESR	Baseline	2.30 ± 0.78	2.23 ± 1.08			
	8 weeks	2.41 ± 0.76	2.24 ± 0.99	0.11 (−0.25 to 0.47)	0.541	0.12
	16 weeks	2.58 ± 0.95	2.16 ± 0.98	0.40 (−0.09 to 0.88)	0.104	0.41
TJC	Baseline	0.31 ± 0.60	0.56 ± 1.41			
	8 weeks	0.44 ± 0.73	0.36 ± 0.93	0.22 (−0.20 to 0.65)	0.292	0.26
	16 weeks	0.44 ± 0.81	0.13 ± 0.35	0.43 (0.01 to 0.85)	0.045	0.69
SJC	Baseline	0.13 ± 0.34	0.25 ± 0.78			
	8 weeks	0.44 ± 0.89	0.50 ± 1.09	0.07 (−0.39 to 0.52)	0.767	0.07
	16 weeks	0.25 ± 0.45	0.33 ± 0.49	0.03 (−0.37 to 0.44)	0.867	0.06
Patient VAS	Baseline	12.75 ± 19.13	26.00 ± 22.97			
	8 weeks	13.75 ± 21.16	23.29 ± 20.40	2.06 (−4.93 to 9.04)	0.551	0.10
	16 weeks	14.38 ± 22.01	28.40 ± 24.82	0.35 (−8.67 to 9.38)	0.937	0.01
Physician VAS	Baseline	2.50 ± 4.32	4.94 ± 9.49			
	8 weeks	7.00 ± 10.48	4.64 ± 5.68	3.65 (−1.68 to 8.99)	0.1714	0.43
	16 weeks	4.38 ± 6.16	3.73 ± 4.89	1.73 (−2.10 to 5.56)	0.3628	0.31
IPAQ	Baseline	1023.2 ± 814.8	2307.0 ± 1713.9			
	16 weeks	1683.1 ± 1720.3	2293.0 ± 2778.4	755 (−737 to 2246)	0.309	0.33
Pedometer (steps/week)	Baseline	34856.4 ± 21271.5	36956.1 ± 20920.2			
	16 weeks	42167.6 ± 24885.4	36432.3 ± 20910.2	7463 (−1950 to 16,876)	0.113	0.32
SF12 PCS	Baseline	46.35 ± 11.19	47.82 ± 9.69			
	16 weeks	46.60 ± 12.92	47.66 ± 9.20	−0.07 (−6.50 to 6.35)	0.982	−0.01
PF	Baseline	48.98 ± 10.60	48.98 ± 8.92			
	16 weeks	46.91 ± 12.27	49.68 ± 9.86	−2.78 (−9.50 to 3.95)	0.405	−0.25
RP	Baseline	50.73 ± 9.10	48.99 ± 8.17			
	16 weeks	47.23 ± 9.99	47.93 ± 8.70	−1.89 (−7.309 to 3.53)	0.482	−0.20
BP	Baseline	51.46 ± 9.04	50.07 ± 7.82			
	16 weeks	50.76 ± 9.82	49.34 ± 9.51	0.18 (−4.45 to 4.81)	0.937	0.02
GH	Baseline	49.92 ± 9.80	53.29 ± 8.80			
	16 weeks	48.71 ± 11.35	51.27 ± 8.76	0.11 (−5.09 to 5.31)	0.581	0.01
SF12 MCS	Baseline	55.58 ± 7.57	57.29 ± 7.63			
	16 weeks	52.88 ± 9.99	55.27 ± 7.90	−0.94 (−5.60 to 3.73)	0.684	−0.10
VT	Baseline	54.53 ± 11.38	56.78 ± 9.72			
	16 weeks	51.15 ± 10.66	55.66 ± 8.70	−3.24 (−8.91 to 2.44)	0.253	−0.33
SF	Baseline	52.68 ± 5.18	50.13 ± 10.96			
	16 weeks	50.14 ± 8.04	49.49 ± 10.16	−1.02 (−6.42 to 4.38)	0.703	−0.11
RE	Baseline	50.39 ± 7.69	50.05 ± 6.97			
	16 weeks	47.68 ± 9.82	51.06 ± 8.70	−3.68 (−8.49 to 1.12)	0.128	−0.40
MH	Baseline	57.10 ± 7.64	55.65 ± 7.49			
	16 weeks	52.03 ± 9.44	54.20 ± 7.34	−2.89 (−8.50 to 2.73)	0.302	−0.34
HAQ-DI	Baseline	0.313 ± 0.342	0.375 ± 0.440			
	16 weeks	0.313 ± 0.368	0.359 ± 0.500	0.02 (−0.12 to 0.15)	0.798	0.05
EQ5D	Baseline	0.84 ± 0.13	0.85 ± 0.13			
	16 weeks	0.87 ± 0.16	0.86 ± 0.12	0.01 (−0.05 to 0.07)	0.697	0.07
FACIT-F	Baseline	12.19 ± 6.36	10.75 ± 6.35			
	16 weeks	13.12 ± 6.76	12.81 ± 5.27	−0.84 (−3.25 to 1.57)	0.479	−0.14

Data are expressed as mean ± SD. CI: confidence interval, DAS: disease activity score, ESR: erythrocyte sedimentation rate, TJC: tender joint count, SJC: swollen joint count, VAS: visual analogue scale, IPAQ: international. physical activity questionnaire, SF: short form, PCS: physical component summary, PF: physical functioning, RP: role physical, BP: bodily pain, GH: general health, MCS: mental component summary, VT: vitality, SF: social functioning, RE: role emotional, MH: mental health, EQ5D: EuroQol 5 dimension, FACIT-F: Functional Assessment of Chronic Illness Therapy-Fatigue

**Fig. 2:**
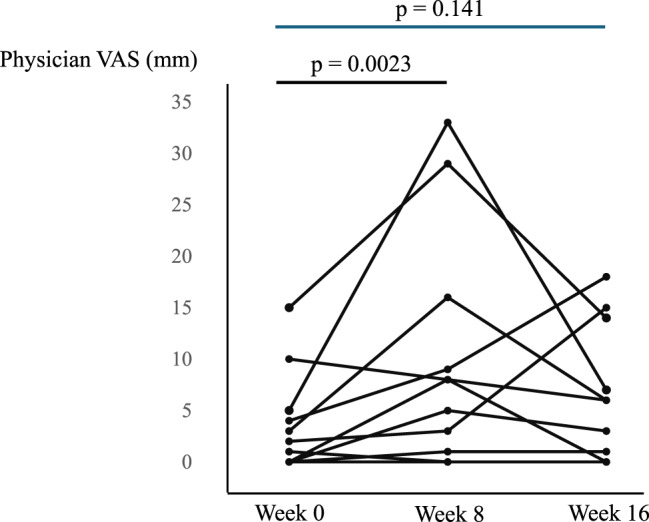
Individual changes in physician VAS in the intervention group. Individual participant values at baseline and weeks 8 (left) and 16 (right) are shown. A transient increase was observed at week 8, with values returning to approximately baseline levels by week 16. P values are presented for descriptive purposes only

A transient increase in the physician visual analog scale (VAS) score was observed in the intervention group at week 8 compared with the baseline (mean change, 4.50; 95% CI, 0.61 to 8.39; standardized effect size, 0.57), with values returning to the baseline by week 16 (Fig. 2).

### Treatment modifications during follow-up

Tapering strategies and treatment modifications during follow-up are summarized in Supplementary Table 3. During the 16-week follow-up period, three participants resumed full-dose TT, including two in the control group (etanercept or baricitinib) and one in the intervention group (abatacept). Methotrexate and prednisolone doses remained stable for all participants. Aside from these cases, no other tapering modifications, dose adjustments, interval changes, or additional disease-modifying therapies were performed during the study period.

All participants were included in the analysis on an intention-to-treat basis. To account for potential confounding related to treatment changes, sensitivity analyses were conducted after excluding participants who resumed full-dose therapy, considered to represent major treatment re-escalation. These analyses yielded results consistent with the primary analysis, indicating that tapering heterogeneity did not substantially influence the exploratory findings.

### Changes in physical activity and exercise adherence

Participants in the intervention group were prescribed three exercise sessions per week for 16 weeks, each lasting approximately 30 to 60 minutes, corresponding to a total of 48 sessions and a mean weekly exercise duration of 90 to 180 minutes. The mean adherence rate was 89.3 ± 20.6%, and all participants achieved the target Borg rating of perceived exertion (RPE 5–6) during the prescribed sessions. Exercise performed outside the intervention was not assessed.

Target exercise intensity for endurance training, assessed using the Borg scale, showed a gradual increase in the intervention group (Supplementary Figure 1). The baseline IPAQ score was lower in the intervention group than in the control group (mean difference, −1283.7 MET-min/week; 95% CI, −2214.0 to −353.5; standardized effect size, −0.96). After adjustment for baseline values, the intervention group demonstrated a modest increase in IPAQ score at week 16 compared with the control group (adjusted mean difference in change, 673.8 MET-min/week; 95% CI, −643.6 to 1991.3), corresponding to a small-to-moderate effect size and partial attenuation of the baseline. Weekly step counts were comparable between groups at baseline and week 16, although a non-significant trend toward increased step counts was observed in the intervention group (Table 2).

### Effects on health-related quality of life

**Fig. 3:**
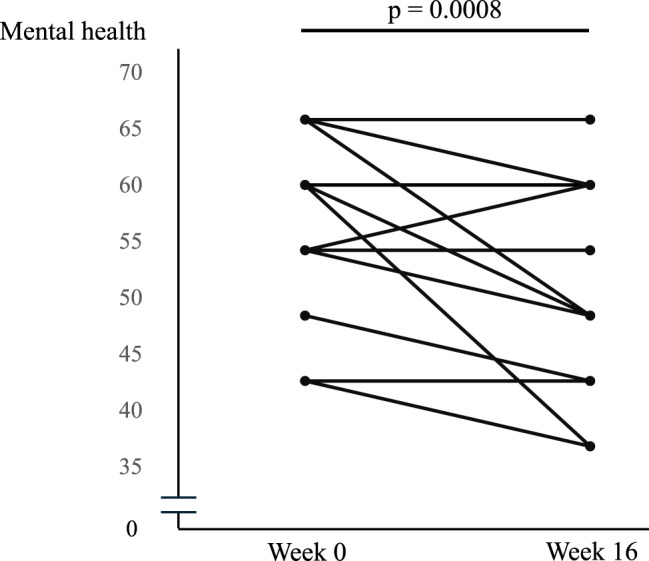
Individual changes in the mental health component of SF-12 in the intervention group. Individual participant values at baseline and week 16 are shown. A downward trend in mental health scores was observed following the exercise intervention. P values are presented for descriptive purposes only

No clear between-group differences in health-related QOL were observed at baseline or after 16 weeks. Within the intervention group, SF-12 mental health (MH) and vitality (VT) scores showed declines over the 16-week period (MH: estimated mean change, −5.07 points; 95% CI, −8.80 to −1.34; standardized effect size, −0.78; VT: estimated mean change, −3.38 points; 95% CI, −6.55 to 0.21; standardized effect size, −0.45) (Fig. 3). In line with these findings, the mental component summary score also showed a decreasing trend (estimated mean change, −2.70 points; 95% CI, −5.59 to 0.19; standardized effect size, −0.46).

Physical component summary scores remained largely unchanged in both groups. HAQ-DI, EQ-5D, and FACIT-F scores showed no clinically meaningful between-group differences or notable changes over time (Table 2).

### Adverse events

No adverse events were observed in either group during the 16-week follow-up period, including musculoskeletal pain, flares, falls, or exercise discontinuation (Supplementary Table 4). For this study, flare was defined as a clinically significant increase in disease activity that required treatment escalation, and considered distinct from loss of remission or LDA without therapeutic intervention.

## Discussion

To the best of our knowledge, this is the first randomized pilot trial performed to evaluate the feasibility, safety, and preliminary effects of individualized ET in patients with RA undergoing TT tapering after achieving remission or LDA. After 16 weeks, no clear between-group differences in disease control were observed, with most participants in both groups maintaining remission or LDA. These findings indicate that ET is feasible and generally safe during TT tapering. Given the pilot design and limited sample size, the results should be interpreted as providing preliminary estimates and methodological insights for future definitive trials, rather than conclusive evidence of clinical efficacy.

At week 8, a transient increase in the physician global assessment score was observed in the intervention group. This temporary change may reflect physiological adaptation to exercise or mechanical stress on the joints during the early phase of the ET program. Importantly, scores returned to baseline by week 16 and were not associated with serious adverse events. Although physical activity levels increased after adjustment for baseline values, mental health and vitality scores declined, suggesting that initiation of ET may impose psychological stress or increase fatigue. Together, these findings underscore the importance of incorporating psychological support, gradual progression of exercise intensity, and careful monitoring when implementing an ET program in RA patients. Nevertheless, the observations require careful consideration, as participants underwent both medication tapering and initiation of structured exercise, potentially resulting in a dual psychological burden. Furthermore, fear of symptom exacerbation during increased physical activity (kinesiophobia) may have contributed to reduced perceived mental well-being in at least some of the participants.

Several factors may explain the absence of a distinct anti-inflammatory effect. The 16-week observation period might have been too short for the biological benefits of exercise to fully manifest. In addition, the type and intensity of the prescribed exercise may not have been sufficient to elicit myokine-mediated anti-inflammatory responses, while remote exercise guidance might have limited adherence and individualized feedback. Furthermore, ongoing TT tapering itself may have contributed to disease instability, thereby attenuating the observable benefits of ET. Together, these findings suggest that ET should be introduced in patients with well-controlled disease activity and that TT tapering should be implemented cautiously, after remission has been confirmed.

Baseline physical activity levels were lower in the intervention group, thus baseline-adjusted analyses were performed to minimize potential regression-to-the-mean effects. After adjustment, ET showed a tendency toward increased physical activity, suggesting that individualized exercise programs can facilitate behavioral change even during medication tapering, which supports the feasibility of ET as an adjunctive intervention in this population. An additional consideration is heterogeneity in TT tapering strategies and treatment modifications during follow-up. Although tapering approaches varied according to drug class and clinical judgment, after excluding participants with major treatment re-escalation, sensitivity analyses yielded results consistent with the primary analysis. Nevertheless, use of a standardized tapering protocol or stratification by therapy type should be considered in future larger trials to better isolate the effects of ET.

This pilot study has several limitations, including small sample size, short observation period, and reliance on remote exercise instruction. As this study was not powered to detect efficacy outcomes, effect estimates should be interpreted with caution. Furthermore, psychological factors, such as fear of movement, motivation, and readiness for exercise, were not assessed, though may have influenced both adherence and mental health outcomes. The relatively long disease duration of the study population is another limitation and may have reduced responsiveness to exercise therapy during treatment tapering. It will be important for future studies to investigate earlier introduction of exercise therapy in patients with early RA. Key strengths of this study include randomized allocation and blinded outcome assessment, which enhance the reliability of the findings. The present results provide important information to guide the design of future large-scale, long-term trials aimed at validating these preliminary findings and optimizing ET protocols for patients undergoing TT tapering.

## Conclusion

Findings from this pilot feasibility study indicate that individualized ET during TT tapering is safe and feasible. No clear between-group differences in maintenance of remission or LDA were observed, however, the study was not powered to evaluate efficacy. The results also provide valuable insights for the design of future large-scale trials. It was concluded that integrated mental health support and optimization of prescribed exercise are important considerations for proper evaluation of the efficacy of ET in RA patients undergoing medication tapering.

## Electronic supplementary material

Below is the link to the electronic supplementary material.

Supplementary material 1

Supplementary material 2

## Data Availability

The datasets used and analyzed for this study are not publicly available, though are available from the corresponding author upon reasonable request.

## Abbreviations

ACPA: anti-citrullinated protein/peptide antibody
BMI: body mass index
BP: body pain
CTLA: cytotoxic T-lymphocyte antigen
DAS: disease activity score
EQ-5D: EuroQol-5-Dimension
ESR: erythrocyte sedimentation rate
ET: exercise therapy
FACIT-: functional assessment of chronic illness therapy-fatigue
GC: glucocorticoid
GH: general health
HAQ-DI: health assessment questionnaire-disability index
IL-6i: interleukin-6 inhibitor
IPAQ: international physical activity questionnaire
JAKi: Janus kinase inhibitor
LDA: low disease activity
MCS: mental component summary
MH: mental health
MTX: methotrexate
PCS: physical component summary
PF: physical functioning
QOL: quality of life
RA: rheumatoid arthritis
RE: role emotional
RF: rheumatoid factor
RP: role physical
SF: social functioning
SF-12: short form-12 survey
SJC: swollen joint count
TJC: tender joint count
TNFi: tumor necrosis factor inhibitor
TT: targeted drug therapy
VAS: visual analog scale
VT: vitality

## Declarations

### Protocol availability

Details of the study protocol are available from the corresponding author upon reasonable request.

### Any other identifying information

There is no identifying information related to the authors, their institutions, funders, or approval committees that might compromise anonymity.
