# Supplementary figures and images for: Personalized exercise therapy during targeted drug therapy tapering in patients with rheumatoid arthritis: a pilot randomized controlled trial
Source: BMC Rheumatol. 2026 May 13;10:57. doi: 10.1186/s41927-026-00646-8 (PMC13340000; doi:10.1186/s41927-026-00646-8)

## Slide 1
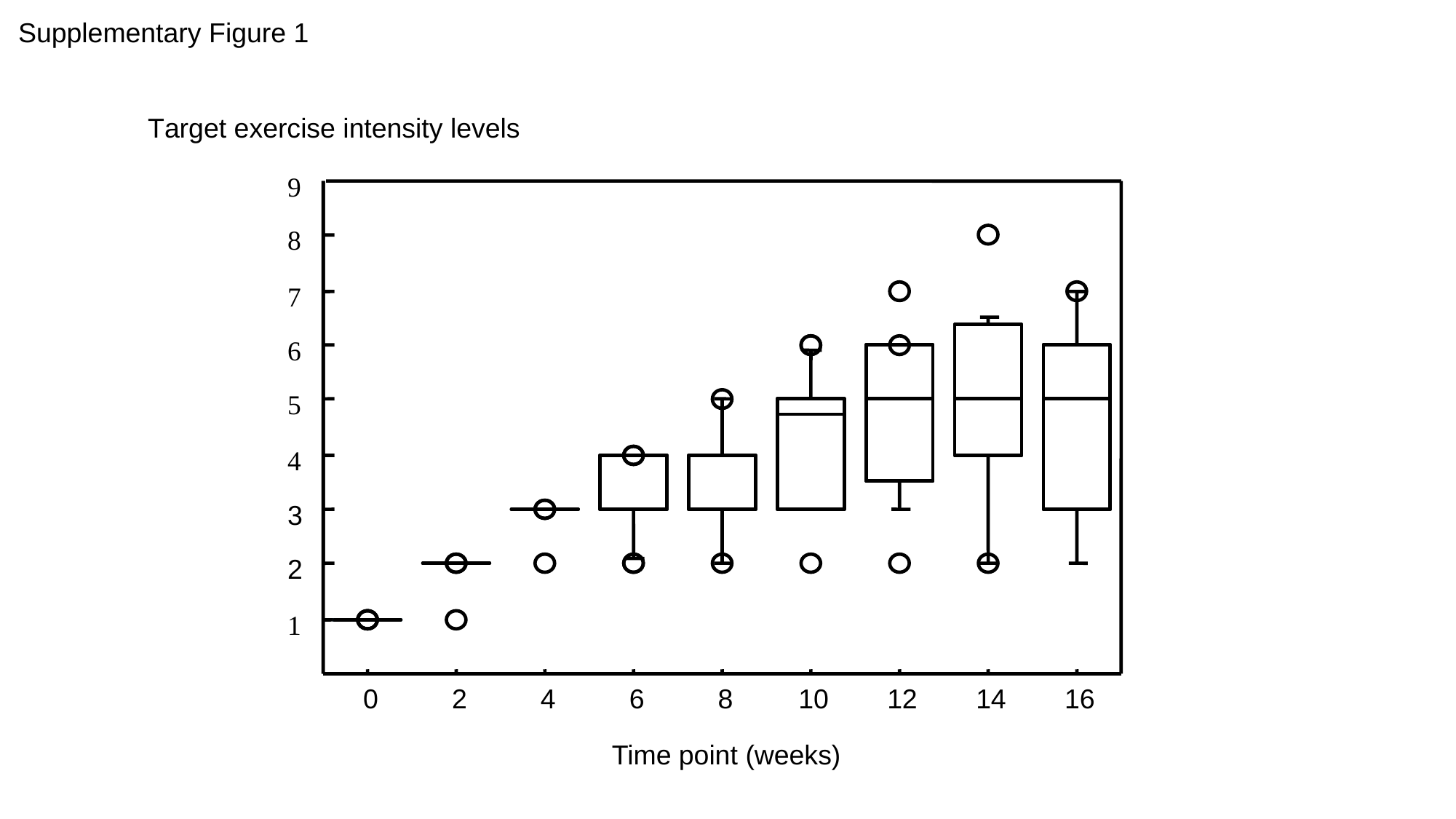

Supplementary Figure 1
Target exercise intensity levels
9
8
7
6
5
4
3
2
1
0
2
4
6
8
10
12
14
16
Time point (weeks)

Supplement: Supplementary file 1 — Supplementary material 1 [file 41927_2026_646_MOESM1_ESM.pptx]
