# Supplementary material for: Personalized exercise therapy during targeted drug therapy tapering in patients with rheumatoid arthritis: a pilot randomized controlled trial
Source: BMC Rheumatol. 2026 May 13;10:57. doi: 10.1186/s41927-026-00646-8 (PMC13340000; doi:10.1186/s41927-026-00646-8)
Supplement: Supplementary file 2 — Supplementary material 2 [file 41927_2026_646_MOESM2_ESM.docx]

Supplementary Table 1. Time schedule for participant enrollment, allocation, interventions, assessments, and visits

|  | **STUDY PERIOD** | | | | | |
| --- | --- | --- | --- | --- | --- | --- |
|  | **ENROLLMENT** | **ALLOCATION** | **POST-ALLOCATION** | | | **FOLLOW-UP** |
| **Time point (weeks)** | -4 | 0 | 2, 4 | 8 | 10, 12 | 16 |
| **Enrollment** |  |  |  |  |  |  |
| Eligibility screening | Ra |  |  |  |  |  |
| Informed consent | E |  |  |  |  |  |
| Allocation |  | Ra |  |  |  |  |
| **Intervention** |  |  |  |  |  |  |
| Exercise program |  | ● | ● | ● | ● |  |
| **Assessment** |  |  |  |  |  |  |
| Baseline variables | E | E |  |  |  |  |
| Outcomes |  |  |  |  |  |  |
| DAS28 |  | E |  | E |  | E |
| Remission/LDA on tapering |  | E |  | E |  | E |
| Physical activity |  | Pt |  | Pt |  | Pt |
| HAQ-DI |  | Pt |  |  |  | Pt |
| SF-12 |  | Pt |  |  |  | Pt |
| EQ-5D-3L |  | Pt |  |  |  | Pt |
| FACIT-F scale |  | Pt |  |  |  | Pt |
| Safety |  |  | Ra | Ra | Ra | Ra |

Ra: research assistant; E: evaluator; Pt: patient; DAS: disease activity score; LDA: low disease activity; HAQ-DI: health assessment questionnaire disability index; SF-12: 12-item short-form health Survey; EQ-5D-3L: European Quality of Life 5 Dimensions 3 Level Version; FACIT-F: functional assessment of chronic illness therapy fatigue

Supplementary Table 2. Methods for tapering targeted drug therapy

| **Mode of action** | **Drug** | **Standard** | | **Tapering** | | | |
| --- | --- | --- | --- | --- | --- | --- | --- |
|  |  |  |  | **Dose reduction** | | **Spacing** | |
|  |  | **Dosage** | **Interval** | **Dosage** | **Interval** | **Dosage** | **Interval** |
| **TNFi** | **IFX** | 3 mg/kg | 1/8 weeks | ≤2 mg/kg | 1/8 weeks | 3 mg/kg | 1 ≥12 weeks |
|  | **ETN** | 25 mg | 2/7 days | - | - | 25 mg | 1/day |
|  |  | 50 mg | 1/7 days | 25 mg | 1/7 days |  | 1 ≥10 days |
|  | **ADA** | 40 mg | 2/week | - | - | 40 mg | 1 ≥3 weeks |
|  | **GLM** | 50 mg | 1/4 weeks | - | - | 50 mg | 1 ≥6 weeks |
|  |  | 100 mg | 1/4 weeks | 50 mg | 1/4 weeks | 100 mg | 1 ≥6 weeks |
|  | **CZP** | 200 mg | 1/2 weeks | - | - | 200 mg | 1 ≥3 weeks |
|  |  | 400 mg | 1/4 weeks | 200 mg | 1/4 weeks | 400 mg | 1 ≥6 weeks |
| **IL-6i** | **TCZ** | 8 mg/kg | 1/4 weeks | ≤5.3 mg | 1/4 weeks | 8 mg/kg | 1 ≥6 weeks |
|  |  | 162 mg | 1/2 weeks | - | - | 162 mg | 1 ≥3 weeks |
|  | **SAR** | 150 mg | 1/2 weeks | - | - | 150 mg | 1 ≥10 days |
|  |  | 200 mg | 1/2 weeks | 150 mg | 1/2 weeks | 200 mg | 1 ≥10 days |
| **CTLA4-IgG** | **ABT** | 125 mg | 1/7 days | - | - | 125 mg | 1/10 days |
|  |  | 500 mg (BW <60 kg） | 1/4 weeks | 250 mg (BW <60 kg） | 1/4 weeks | 500 mg (BW <60 kg） | 1 ≥6 weeks |
|  |  | 750 mg (BW 60-100 kg) | 1/4 weeks | 500 mg (BW 60-100 kg) | 1/4 weeks | 750 mg (BW 60-100 kg) | 1 ≥6 weeks |
|  |  | 1000 mg (BW 100 kg) | 1/4 weeks | 750 mg (BW >100 kg) | 1/4 weeks | 1000 mg (BW >100 kg) | 1 ≥6 weeks |
| **JAKi** | **TOF** | 5 mg | 2/day | - | - | 5 mg | 1/day |
|  | **BAR** | 4 mg | 1/day | 2 mg | 1/day | - | - |
|  | **PEF** | 150 mg | 1/day | 100 mg | 1/day | - | - |
|  | **UPA** | 15 mg | 1/day | 7.5 mg | 1/day | - | - |
|  | **FIL** | 200 mg | 1/day | 100 mg | 1/day | - | - |

TNFi: tumor necrosis factor inhibitor; IL-6i: interleukin-6 inhibitor; CTLA: cytotoxic T-lymphocyte antigen; JAKi: Janus kinase inhibitor; IFX: infliximab; ETN: etanercept; ADA: adalimumab; GLM: golimumab; CZP: certolizumab pegol; TCZ: tocilizumab; SAR: sarilumab; ABT: abatacept; TOF: tofacitinib; BAR: baricitinib; PEF: pimicotinib; UPA: upadacitinib; FIL: filgotinib, BW: body weight

Supplementary Table 3. Tapering modifications and treatment resumption during follow-up

| Group | Participant ID | Drug | Change | Time of change (week no.) |
| --- | --- | --- | --- | --- |
| Control | 15 | Etanercept | Resumed standard dose | 0 |
|  | 24 | Baricitinib | Resumed standard dose | 8 |
|  | 15 | Etanercept | Resumed standard dose | 0 |
| Intervention | 26 | Abatacept | Resumed standard dose | 8 |

Supplementary Table 4. Adverse events noted in 16-week follow-up examination

| Adverse event | Intervention group (n=16) | Control group (n=16) |
| --- | --- | --- |
| Musculoskeletal pain | 0 | 0 |
| Flare | 0 | 0 |
| Fall | 0 | 0 |
| Exercise discontinuation | 0 | 0 |
